# A prospective cohort study to evaluate peridomestic infection as a determinant of dengue transmission: Protocol

**DOI:** 10.1186/1471-2458-12-262

**Published:** 2012-04-02

**Authors:** Ruth Aralí Martínez-Vega, Rogelio Danis-Lozano, Jorge Velasco-Hernández, Fredi Alexander Díaz-Quijano, Mariana González-Fernández, René Santos, Susana Román, Jorge Argáez-Sosa, Miguel Nakamura, José Ramos-Castañeda

**Affiliations:** 1 Centro de Investigaciones sobre Enfermedades Infecciosas (CISEI), Instituto Nacional de Salud Pública (INSP), (Av. Universidad 655), Cuernavaca, (62100), México; 2 Departamento de Control de Vectores, INSP, (19 Calle Poniente, esquina 4ta Norte s/n), Tapachula, (30700), México; 3 Programa de Investigación en Matemáticas aplicadas y computación, Instituto Mexicano del Petróleo, (Eje Central Lázaro Cárdenas Norte 152), Ciudad de México, (07730), México; 4 Organización Latinoamericana para el Fomento de la Investigación en Salud (OLFIS), (Av. Búcaros No. 2-108 Laureles C-60), Bucaramanga, (680005), Colombia; 5 Centro Regional de Control de Vectores de Cuautla, Servicios de Salud de Morelos (SSM), (Callejón Borda No.3), Cuernavaca, (62000), México; 6 Subdirección de Geografía Médica y Sistemas, INSP, (Av. Universidad 655), Cuernavaca, (62100), México; 7 Centro de Investigación en Matemáticas (estadística aplicada), Universidad Autónoma de Yucatán, (Periférico Norte Tablaje 13615), Mérida, (97110), México; 8 Departamento de Probabilidad y estadística, Centro de Investigación en Matemáticas, Jalisco S/N, Guanajuato, (36240), México

**Keywords:** Dengue, Transmission, Peridomestic, Cohort, Immunity

## Abstract

**Background:**

Vector control programs, which have focused mainly on the patient house and peridomestic areas around dengue cases, have not produced the expected impact on transmission. This project will evaluate the assumption that the endemic/epidemic transmission of dengue begins around peridomestic vicinities of the primary cases. Its objective is to assess the relationship between symptomatic dengue case exposure and peridomestic infection incidence.

**Methods/Design:**

A prospective cohort study will be conducted (in Tepalcingo and Axochiapan, in the state of Morelos, Mexico), using the state surveillance system for the detection of incident cases. Paired blood specimens will be collected from both the individuals who live with the incident cases and a sample of subjects residing within a 25-meter radius of such cases (exposed cohort), in order to measure dengue-specific antibodies. Other subjects will be selected from areas which have not presented any incident cases within 200 meters, during the two months preceding the sampling (non-exposed cohort). Symptomatic/asymptomatic incident infection will be considered as the dependent variable, exposure to confirmed dengue cases, as the principal variable, and the socio-demographic, environmental and socio-cultural conditions of the subjects, as additional explanatory variables.

**Discussion:**

Results indicating a high infection rate among the exposed subjects would justify the application of peridomestic control measures and call for an evaluation of alternate causes for insufficient program impact. On the other hand, a low incidence of peridomestic-infected subjects would support the hypothesis that infection occurs outside the domicile, and would thus explain why the vector control measures applied in the past have exerted such a limited impact on cases incidence rates. The results of the present study may therefore serve to reassess site selection for interventions of this type.

## Background

Dengue, the most frequent vector-borne viral disease in the world, constitutes a public health problem in tropical and subtropical countries [1]. In its 2008 and 2009 reports to the World Health Organization, Mexico reported 31,154 and 55,363 confirmed dengue cases (24.42 cases per 100,000 inhabitants), with 24 and 96 fatality cases, respectively.

Dengue affects the productivity of countries significantly, and generates losses that are as important as those caused by tuberculosis, Chagas, leishmaniasis, hepatitis and malaria, among others [2-5]. For instance, the total cost of the Cuban epidemic in 1997 ascended to 10,251,539 USD, taking into account the money invested in vector control and patient care [6]. Moreover, the direct economic impact of the disease is compounded by the disability-adjusted life years (DALYs) [2,4]. For example, in Puerto Rico, from 1984 to 1994, dengue caused an average of 658 DALYs per million inhabitants per year, with a maximum of 2,153 DALYs per million inhabitants estimated for 1994. The greatest burden of disease was attributed to dengue fever [4].

It has been established that Dengue virus (DENV) transmission involves a man-vector-virus pathway, but the characteristics and dynamics of the related factors have not been fully determined [7]. Firstly *Aedes aegypti *and secondly *Aedes albopictus *mosquitoes have been found to be the leading dengue vectors in the world. However, the virus has also been isolated from species such as *Ae. albifasciatus *and *Ae. polinensis *[8].

The dengue endemic/epidemic cycle is maintained by the vector through mosquito-man- mosquito transmission. On contracting the virus, mosquitoes remain infected and asymptomatic for the remainder of their lives. Human beings are both the definitive host and the reservoir of the virus. A female mosquito feeds on a number of humans during the day. Once it has bitten an infected individual, it incubates the virus for a period of 7-14 days, and then starts to infect healthy individuals through its bite. In humans, DENV incubation extends over a period of 3 to 14 (7 on average) days. The actual infectious stage, or time span during which the diseased person spreads the infection to a vector, commences one to two days before the onset of symptoms, at which time high levels of viremia appear and continue to develop throughout a febrile cycle of 2 to 10 days [9].

The DENV infection spectrum covers symptomatic cases (dengue fever, dengue hemorrhagic fever, dengue shock syndrome and death), as well as asymptomatic cases, which, in most studies, represent over half of total infections [10-23]. It has been proposed that the high frequency of asymptomatic cases may be contributing to the maintenance of human transmission despite the reduced number of vectors during inter-epidemic periods [24]. Likewise, as the level of viremia required to produce clinical manifestations is currently unknown [25] and asymptomatic viremic cases of dengue have been reported [14,26], the asymptomatic cases may constitute a determinant of persistent dengue transmission. Being at least as frequent as the symptomatic cases, and not having been combated by preventive measures of any sort, these may well be a source of vector infection, and could thus be generating a significant impact on the spread of dengue among different populations.

Given the absence of a DENV-specific vaccine or antiviral treatment, health services have focused their prevention efforts on vector control. Government vector control programs offer a number of partially successful examples. For instance, in 1965, a PAHO campaign against urban yellow fever (consisting of DDT and malathion spraying) eradicated *Ae. aegypti *from America and, collaterally, attenuated dengue. However, the program was discontinued in the early 1970s and America has been reinfested with *Aedes*. A second partially successful example occurred in Singapore in 1973, when a government program based on entomological surveillance, larval breeding-site reduction, education and the enforcement of legislation to control *Ae. aegypti *and *Ae. albopictus*, slashed the House Index (HI) from 50% to 2% and curbed dengue prevalence. Notwithstanding, at 15 years, dengue incidence has intensified, without, however, hoisting the HI [27].

Cuba provides yet another example of success: subsequent to its 1981 epidemic, the Cuban government conducted a vector eradication campaign where both vector and dengue were eliminated from the island for 15 years. After 1997, however, despite a stable 1% HI, the virus resurged in certain localities, probably due to a drop in herd immunity and transmission by international travelers. In the two aforementioned cases, government programs have achieved long-term dengue control as a result of the countries' political will and geographic conditions.

At the government level, vector control measures have concentrated on peridomestic dengue areas. For instance, the Mexican National Health Standard obligates the state health services to execute larval control and dejunking campaigns permanently, as well as nebulization and fumigation procedures during peak transmission periods, targeting the domiciles and peridomestic vicinities of dengue cases detected through epidemiological surveillance. Nevertheless, the results have fallen short of expectations.

In this regard, a literature review of Esu E, et al [28] was conducted to evaluate the effectiveness of peridomestic space fumigation for reducing dengue transmission and yielded no conclusive evidence of reduction through this measure: only one of the 15 studies reviewed reported an impact on the disease (a reduction of case incidence during the four weeks subsequent to the intervention). In this instance, however, the application of insecticides was coupled with anti-breeding campaigns. While 13 studies reported initial cuts in the entomological indices, most cases achieved only an ephemeral impact. As a result, the authors of the review sustain that a reduction in entomological indices does not necessarily imply a reduction in transmission, due to the presence of additional factors relating, for instance, to herd immunity, population density, circulating serotype and a number of other environmental variables [28]. Further potential factors include a rise in extra-domiciliar transmission, changes in vector surveillance policies and migratory movements [27].

Moreover, as regards vector density and its relation to dengue, a study in Thailand reported a negative correlation between the Breteau Index and dengue incidence [24]. Another study in Brazil revealed not only that the peak case incidence period was not preceded by a rise in vectors in the locality, but also that mosquito density was low when the occurrence of cases was the highest, thus suggesting that infection took place outside the case domiciles (schools, workplaces, etc.) [29]. Other transmission-related factors have been contemplated, such as: age, low educational and income levels, the number of occupants per room, houses -not apartments- as dwellings, study and work sites outside the home, and subject movement patterns [23,24,30-32].

Regarding intra and peridomestic transmission, a study in Thailand reported that only 13 out of 180 families with subjects hospitalized for dengue presented more than one hospitalized case in their households [33]. Another study in Puerto Rico evaluating the occurrence of infection with a four-month follow up found that out of the 189 sample households (with two or more persons residing with an incident case) 95 presented at least one infected individual during follow up, and only 50 of these indicated two or more infected individuals [23].

So far, three studies reported in the literature have evaluated infection in the vicinity of pediatric index cases (through cluster sampling). The first, conducted in Indonesia with a 14 day follow up, included persons living with and neighbors residing within a 10-meter radius of the cases. The results indicated a recent-infection rate of 24.5% (192/785 cases, 17 of which consisted of post-enrollment infections). The index case serotype was analyzed and compared to that of the infected individuals in the surrounding areas: it proved identical in four out of the seven clusters studied, but different in three [26]. The second study, conducted in Thailand with a 15-day follow up, sampled children aged six to 15 years residing within a 100-meter radius of the index cases' dwellings. Twelve positive clusters (with an index case) and 22 negative clusters (with no index case) were analyzed, yielding an incidence rate of 12.4% in the first and 0% in the second (with an attributable risk of 10.4% and a CI of 95% 1-19.8%) [13].

Thirdly, a recent study conducted in Nicaragua with a 14-day follow up reported infection in 2.4% (10/414) of the subjects in the 18 positive clusters (composed of individuals aged two and older residing with or within a 50-meter radius of the confirmed dengue index cases) and 2.5% (2/81) of the subjects in the four negative clusters (composed of persons residing with and neighbors of dengue-negative febrile cases) studied. Additionally, the viral sequences of three index cases and four contacts were analyzed, with the sequence of only one contact diverging from that of its index case [10].

In sum, the relationship among the dengue transmission pattern, the incidence of the disease and the density of *Ae. aegypti *is still unclear. Vector-control programs targeting the peridomestic vicinities of cases have not achieved the desired results, and the causes are unknown. They may include inadequate insecticide application, vector resistance to the insecticides applied, non-continuity of the measures implemented and the fact that transmission is not occurring -or is occurring less than expected- within the peridomestic areas.

Hence, identifying the relationship between exposure to a dengue case in its peridomestic vicinities and the frequency of new infections would help to shed light on the dengue-transmission pattern in endemic urban areas. Additionally, the results of this study would both contribute to improve control activities with the purpose of achieving a greater impact on the burden imposed by dengue, and help to give the resources destined for vector control a more efficient use. This is particularly critical in the dengue-endemic areas where budgets for interventions of this type are restricted.

## Methods/Design

### Objectives

The general objective of the present study is to assess the relationship existing between a clinical case of dengue identified under the epidemiological surveillance system and the DENV infection incidence among the subjects residing in the domicile and peridomestic areas of such case.

In order to achieve this, the frequency of DENV infection will be determined in subjects both exposed and not exposed to confirmed dengue cases in their vicinities (index cases). The relationship between the incidence of infection and several socio-demographic, sociocultural and environmental variables will be evaluated. A multivariate model susceptible of explaining the incidence of DENV infection in the area will be constructed. And the extent to which the estimated vector density explains the relationship between the incidence of infection and peridomestic exposure to a dengue case will be assessed.

### Design and population

A prospective cohort study will be conducted in a dengue-endemic urban area with subjects aged five and above who reside in the Tepalcingo and Axochiapan localities and authorize their inclusion in the study. Individuals, for whom follow up is impossible, will be excluded.

### Study area

The towns of Tepalcingo and Axochiapan (seat of the municipal government) are localities

belonging to the third sanitary jurisdiction of state of Morelos. Tepalcingo (N18° 35' 47", W98° 50' 37") rises to 1,160 meters above sea level, covers an area of 4,062,478 m^2^, and has a population of 12,053; Axochiapan (N18° 30' 9", W98° 45' 14") rises to 1,030 meters above sea level, covers an area of 8,967,290 m^2^, and has a population of 16,255, according to the 2010 census [34].

### Variables

**Dependent variable: **recent (symptomatic or asymptomatic) DENV infection confirmed through paired serology.

**Main independent variable: **exposure to an "index case--IC," that is, a dengue case identified through the jurisdictional epidemiological surveillance system, and confirmed through the algorithm applied by the Health Services of Morelos (abbreviated to *SSM *in Spanish). Under the *SSM *algorithm, serum is tested with NS1 ELISA during the first five days of febricity. If the results are negative, IgM ELISA is applied, and if those are negative as well, a quantitative IgG test is performed. Additionally, culturing or RT-PCR analyses are performed on 10% of the confirmed cases to obtain the circulating serotypes. Where the blood samples are drawn on day six of febricity or thereafter: first, an IgM measurement is performed, and, if the results are negative, a quantitative IgG test is applied as well.

**Potential confounding variables: **age, gender, mobility (over the past 15 days, evaluated by means of a questionnaire), human population density and vector-reduction interventions executed by the participating subjects and/or *SSM*.

**Other independent variables: **educational level, occupation, household characteristics, estimated *Ae. aegypti *abundance and their infection with DENV. The circulating viral serotype will be monitored in an IC subsample, although changes are not expected to occur due to limited sampling space and time. Although climatic variables are likely to be identical in the exposed and non-exposed groups, temperature, rainfall and humidity will be monitored on the basis of data provided by either *SSM *or the State Commission for Water and the Environment (abbreviated to *CEAMA *in Spanish).

### Sample size

**Figure 1:**
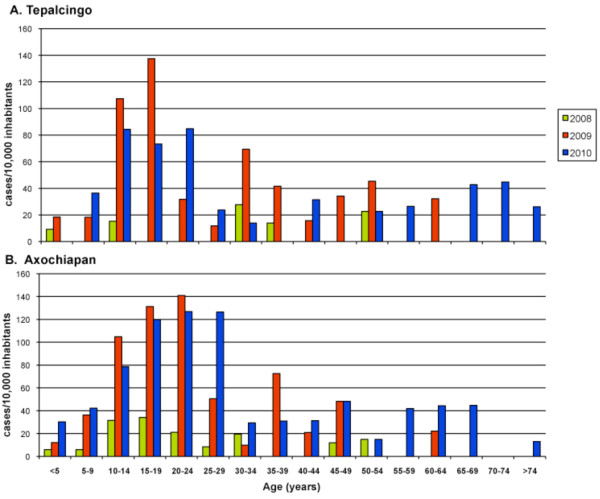
**Dengue incidence per 10,000 inhabitants per year, according to age, Tepalcingo and Axochiapan, Morelos, México**.

According to epidemiological data collected recently (2006-2010) in Tepalcingo and Axochiapan, local DENV incidence averages 44.1 and 46.5 notified confirmed cases/10,000 inhabitants respectively, and afflicts all age groups (Figure 1) (data provided by *SSM*). However, given the presence of significant endemic-area incidence variation, the sample calculation for the study is based on an incidence of 20 notified cases per 10,000 inhabitants per year.

**Figure 2:**
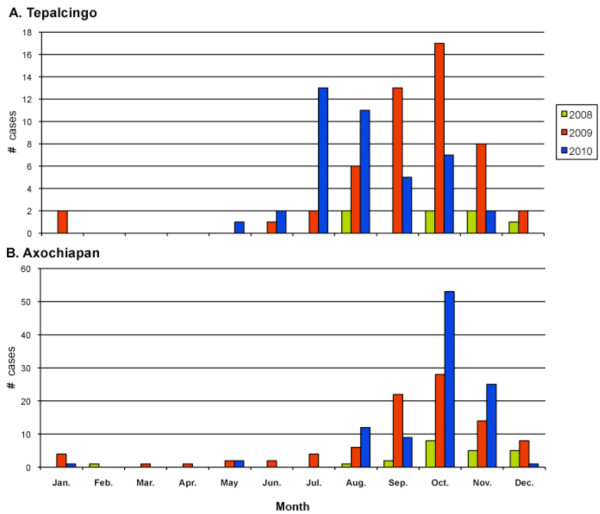
**Monthly distribution of confirmed dengue cases in Tepalcingo and Axochiapan**.

Given that febrile dengue cases are underreported in endemic areas (4 non-reported cases per reported case) [35], the febrile case incidence in Tepalcingo and Axochiapan is expected to reach only 100/10,000 inhabitants. Further, bearing in mind that at least 50% of infections are asymptomatic [10-23], the infection rate in the Tepalcingo and Axochiapan endemic areas is expected to average a minimum of 2% annually. With 80% of infections estimated to occur during the study evaluation and follow-up months (Figure 2), the DENV-infection incidence in the non-exposed group is expected to represent 1.6%.

Considering a statistical power of 80%, a confidence level of 95%, an incidence of 1.6% in the non-exposed group, a 1:1 exposed:non-exposed group relation, an estimated Relative Risk (RR) of 3 and a loss of 10% during follow up, a total of 1,178 subjects will need to be evaluated. Calculations were performed with the Stata 9.2 SE software.

### Sampling, training and follow up

The Morelos state dengue surveillance system will be used to detect the ICs, and these will be visited at home for a standardized interview including questions on socio-demographic and mobility variables.

#### Exposed group

All individuals aged five and above residing in the ICs' domiciles will be invited to participate in the study. Those who accept will be applied a standardized interview with variables regarding their socio-demographic and mobility status, their household characteristics, and the presence of symptoms over the past 15 days (every household will be GPS referenced). Additionally, initial 10-ml blood samples (baseline samples) will be collected from the subjects, and entomological surveys will be conducted in their homes, during which mosquito larvae and pupae will be actively sought within all the containers in the properties, according to the Focks and Pan American Health Organization methodology recommendations [36,37]. Each container will be classified under the EA1 entomology format of the Mexican Ministry of Health. A two-person team will gather samples. Each team will collect all the larvae/pupae present in the small containers (of less than 5 liters). The larvae/pupae samples in the larger containers will be collected by net sweeping each container three times. Subsequently, all larvae/pupae will be dispatched alive to the National Institute of Public Health (*INSP*) insectary for classification and DENV-infection evaluation of the emerging mosquitoes.

Three months (± 7 days) after the initial evaluation, a follow-up visit will be made to the domicile of each IC to: inquire about the onset of any symptoms during the preceding week, take a second blood sample of 6 ml (follow-up sample) and conduct another household entomological survey.

Between the baseline and the follow-up evaluation, active monitoring will be carried out by means of a weekly telephone call, to ascertain whether any febrile events have taken place among the participants. During two days, five phone call attempts will be made at one-hour intervals, but if telephone contact proves impossible, the subjects will be visited at home. Passive monitoring will also be carried out by consulting the *SSM *records to check whether any participants have been reported as suspected dengue cases by the local healthcare centers.

**Figure 3:**
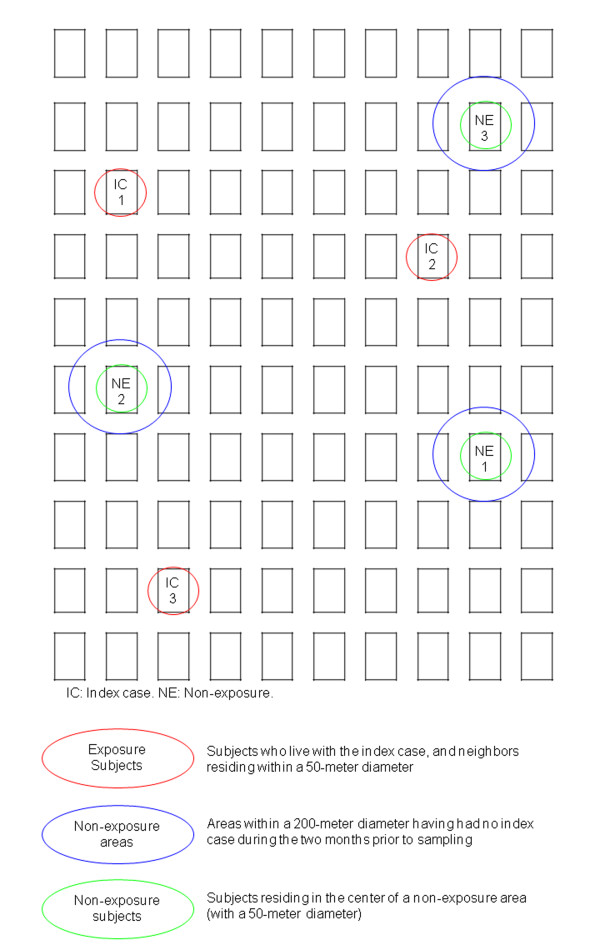
**Sampling of exposed and non-exposed subjects**.

In a 50 meters area next to IC house (that is, inside a circular area with a 25-meter radius) four houses will be invited to participate (Figure 3). The subjects who accept to participate in the study will be applied the same questionnaire, blood sampling and infested-container inspection procedures as those previously applied to the persons residing with the ICs,

It is expected that a total of 15-20 exposed subjects will be enrolled per IC. Of these, three are anticipated to be intradomiciliar, and the rest, 12-17 individuals, peridomestic residents. According to least information available (2005 census), in the study area there are five occupants on average per household. In terms of our study, one of these will be the IC, and another will either not accept to participate or be under five years and therefore ineligible for the study.

#### Non-exposed group

The same week the exposed group is enrolled, sampling will be carried out in those areas that both have not presented ICs in the past two months and are situated within 200 meters of the ICs (the vector flight range reaches a maximum radius of 100 meters) [13]. In a 50 meters circle centered on the first house that at least one person agree to participate other four houses will be to invite to participate as we described for the exposed group (Figure 3). Those who accept will be evaluated in the same manner as the exposed-group subjects. It is expected that 15-20 non-exposed individuals will be included per group of 15-20 exposed individuals. To achieve the calculated sample size, approximately 40 ICs will be evaluated such that 589 exposed and 589 non-exposed subjects may be included in the study.

Furthermore, to avoid oversampling geographic areas, a simulation study was conducted as a means of determining the approximate minimum size required for the study area to ensure with high probability, 40 exposure and 40 non-exposure sections with a diameter of 50 meters each (that is, without overlapping surfaces). Under the simulation study, it was considered that infected individuals turn up randomly throughout the area and that, as a result, the determination of their control sites is also entirely random. This simulation scenario is conservative, as it represents the least favorable situation for producing non-overlapping circles; the true pattern of infection almost surely gives rise a more suitable sampling scheme. According to the simulation procedure, Tepalcingo and Axochiapan offers an adequate study area, as it allowed for obtaining 40 infected subjects and their corresponding 40 cohorts in 100% of the exercises.

### Dengue diagnosis in the subjects (outcome)

Diagnoses will be confirmed by means of paired DENV-specific IgM and IgG ELISA (baseline and follow-up samples) (Panbio Cat No. E-DEN02G and E-DEN01M). Initial IgM detection will be performed on paired samples from each subject, and will be followed by paired IgG tests if results are inconclusive.

Colorimetric readings will be performed with a microplate reader in accordance with the supplier instructions on the diagnosis box. Interpretations will be made with a cutoff value of 0.200 (pre-established with samples from both healthy and diseased individuals in the area), Values equal to or higher than cutoff will be considered as positive; those under cutoff will be considered as negative.

Events covering any of the following conditions will be considered as confirmed **recent DENV infections: **(1) seroconversion (presence of negative antibodies in the baseline and positive antibodies in the follow-up sample); (2) quadriplication of antibody titles between the baseline and the follow-up sample, and (3) availability of a viral isolation result indicating a positive RT-PCR or NSI during the three follow-up months because the subject has developed a febrile manifestation and has attended *SSM *for consultation [38].

Subjects will be considered to be without **recent DENV infection **if the results from their DENV-specific IgM and/or IgG tests are negative, or the matched study samples do not indicate a rise in antibodies.

#### Estimated abundance of mosquitoes

**Figure 4:**
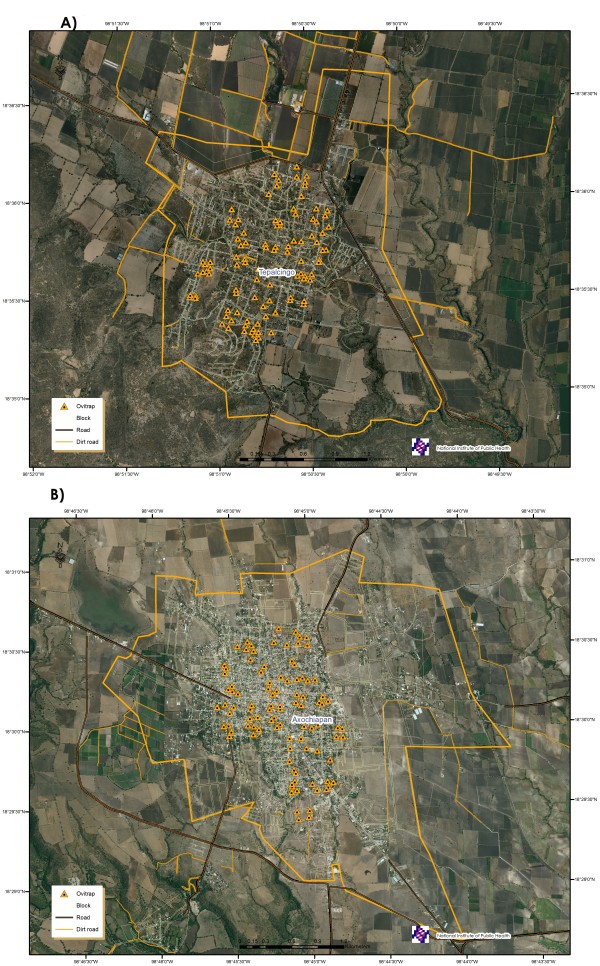
**Location of ovitraps in Tepalcingo(A) and Axochiapan (B), 2011**.

One direct and one indirect estimation will be implemented. The latter will apply data from the entomological surveillance system based on the use of *SSM *ovitraps, which consists in one-year weekly revisions of 100 ovitraps distributed throughout 25 neighborhood blocks within the locality (one ovitrap for each side of the block) (Figure 4). The relative abundance of vectors in the participating dwellings will be estimated with the information obtained from all ovitraps, based on a surface that interpolates the abundance values of the sample sites correlating with suitable covariates at hand over the whole study area, such as solar radiation, construction and vegetation densities, altitude, etc. The determination of the specific interpolation method will depend on both the type of data obtained during sampling and the type and relevance of covariates available over the study area.

The information gathered during the baseline and follow-up surveys of all the participating households will serve directly to calculate the positive-container index (number of infested containers/number of water containers inspected) and the Breteau index (number of positive containers /number of households inspected) for each IC-exposed and non-exposed group.

#### Monitoring Ae. aegypti DENV infection

Not only the mosquitoes that emerge from the larvae/pupae collected at the dwellings of the participating subjects will be analyzed, but also those that emerge from the eggs ovitrapped within a 200-meter radius of the dwellings and collected during the specific IC-enrollment week, the two previous weeks and the subsequent week.

Viral isolation will be performed by culturing mosquito C6/36 (*Ae. albopictus*) cells. Groups of 5-10 mosquitoes will be formed and macerated. The macerate will then be centrifuged, the supernatant will be filtered, and 50 μl of the resulting fluid will be drawn to inoculate the C6/36 cells. These will be incubated at 28°C for approximately 8 days. Then, the cultures will be centrifuged, the cell package will be suspended in PBS, and the suspension will be deposited in five fluorescence slides to be air-dried for one hour and then fixed with -20°C absolute acetone. Next, the preparations will be rehydrated in PBS. First, screening dye (direct immunofluorescence) will be applied. For this purpose, one of the five slides will be incubated with an anti-flavivirus antibody conjugated to fluorescence (direct conjugation, pool of human DENV-positive ELISA-tested sera). Afterwards, it will be washed in PBS and mounted in glycerol-PBS for reading.

In the positive samples, the serotype will be identified by incubating the remaining slides with specific monoclonal serum antibodies and developing the interaction with an anti-IgG antibody from a mouse conjugated to Alexa 488. Next, the preparations will be washed in PBS and incubated with the diluted G-Alexa anti-mlg antibody. Lastly, the preparations will be washed once more in PBS and mounted in glycerol-PBS for reading.

#### Geographic Information System (GIS)

Geographic data on the study area, neighborhoods, blocks, schools, ovitraps, roads, satellite images and contour lines will be compiled and consolidated. The dengue cases reported to the Sole Dengue Epidemiological Surveillance Platform (*Plataforma Única de Vigilancia Epidemiológica de Dengue*) during the study period will be geo-referenced with GPS (Global Positioning System) devices. ArcGIS10 software will be used to develop the GIS application. The aforementioned data will be consolidated under the GIS, and 200- and 50-meter buffers will be traced to determine which households will be contemplated under the follow-up survey. Both the demographic and survey data -including the Breteau Index- will be matched to the study blocks.

#### Data collection and storage

To collect data, an online entry and follow-up system will be developed and pilot tested. The system will include formats for: the screening exercise, the subject baseline survey, the household baseline evaluation, the telephone-follow-up activity, the subject follow-up survey and the household follow-up evaluation. Formats will encompass socio-demographic, mobility and health status variables, among others. The system will validate the information automatically as data is entered, and avoid duplication of variables that are common among the subjects, thus reducing the time and margin of error for data entry. All the information will be stored in a central database where input from the Dengue Epidemiological Surveillance Platform will later be incorporated.

### Statistical analysis

Initially, a description of the population will be formulated using exploratory graphical analysis, frequencies for the categorical variables, and central tendency measures (means and medians, depending on the distribution) as well as dispersion measurements (standard deviation and range) for the continuous variables. Additionally, the incidence of infection will be determined in the exposed and non-exposed groups and the RR will be calculated.

Subsequently, with recent DENV infection as the dependent variable, an appropriate bivariate analysis will be conducted to assess whether an association exists. Of course, the specific method of analysis will depend on properties of the data and validation of assumptions. But possible methods include the chi-square test (for the categorical variables) and either Student's t or the Mann-Whitney test (for the continuous variables). Variables presenting *p *< 0,20 in the bivariate analysis will be evaluated under a binomial regression analysis or an alternate model to obtain a multivariate model susceptible of explaining the DENV-infection incidence; variables presenting *p *< 0,05 or altering the principal variable estimate by 10% or more will remain in the model.

The model will not include the estimated vector density variables, since they are part of the causal mechanism underlying the principal variable association (IC exposure - new infections). However, once the explanatory model has been adjusted to the confounding variables and established, the estimated vector abundance measures will be incorporated (each one separately), with the view of identifying to what extent the vector component might explain the principal variable association. For this purpose, two variables will be considered: mosquito abundance estimated indirectly through ovitraps, and vector density estimated directly by measuring the larvae in the homes of the participating subjects. The analysis will be performed with the Stata SE 10 statistics package, including any necessary diagnostic checks.

### Ethical considerations

The present study is a minimal-risk research project approved by the Ethics Commission of the National Institute of Public Health (*CI: 986, No.1032*). Each subject will be requested a written informed consent for her/his participation in the study. If the subject is a minor (from five to 17 years of age), authorization will be sought from a parent or legal representative. However, if the subject is seven or older, her/his assent will be requested as well.

## Discussion

The development of optimal and successful programs for dengue surveillance and control requires an understanding of where subjects are infected. The scientific evidence derived from this study will make it possible to discern whether dengue transmission occurs in or outside the peridomestic areas of infected cases. Results indicating a high infection rate among the subjects exposed to symptomatic dengue cases would support implementing vector-control measures in the domiciles and vicinities of the cases, and would suggest the need to evaluate alternate causes for insufficient program impact, causes such as inadequate space nebulization techniques and vector resistance to applied insecticides.

However, results indicating that subjects are infected in the peridomestic areas of symptomatic dengue cases at a frequency that is equal or inferior to that of subjects infected in areas not exposed to symptomatic cases, would support the hypothesis that infection occurs outside the domiciles, and would explain why the anti-vector measures applied in the state of Morelos have proved ineffective. These findings would be used both to reassessing and redirecting the selection of sites for prevention and control interventions, also to designing a new proposal for vector-control programs.

An alternate explanation for non-association between IC exposure and new dengue infections may reside in the fact that the "non-exposed" groups could have actually been exposed, but to cases undetected by the epidemiological surveillance system. This finding would call into question the strategy followed by the epidemiological surveillance system to detect and notify cases, which, in themselves, constitute the basis for targeting the community prevention and control interventions implemented by the health authorities.

One of the strengths of the present study refers to its exhaustive (IgM and IgG) diagnostic algorithm which, based on paired samples to confirm recent DENV infection, facilitates an adequate classification of the disease -both symptomatic and asymptomatic- among study subjects. This is highly relevant considering that the immune status of the population is as yet unknown, and could account for different immunological response patterns (relating to primary and secondary infection).

A further strength resides in the prospective detection of asymptomatic-infection subjects, considering that few studies have centered their observation on cases with no clinical manifestations, the case-environment interaction or the participation of cases in the transmission chain. This information is fundamental for assessing the impact that will be achieved by the DENV vaccines currently being piloted in several Latin American countries, including Mexico. It will also serve to assess the minimum coverage required to obtain a herd-immunity effect on the population.

As it is impossible to monitor and ascertain vector density in the domiciles of the participating subjects prior to and during follow up, it will be estimated according to the data from the ovitrap-based *SSM *surveillance program (a permanent but indirect measurement). Additionally, vector index inspections will be performed during visits to the subjects' dwellings (a direct but single time measurement). Due to the difficult logistics involved in the capture of adult Aedes, mosquito infection will be analyzed in the individuals that emerge from the eggs collected through the ovitraps closest to the subjects' dwellings and from the larvae/pupae collected at the dwellings themselves. The two aforementioned limitations may bias the information obtained on the vector-density and vector-infection variables. And the reduced measuring precision may hamper the capacity to evaluate the relevance of these variables as explanatory components of the principal association (IC and infection incidence).

Another limitation refers to the subject-mobility-measurement procedure. Performed by way of a questionnaire (subjective and retrospective), it entails a potential memory bias. Nonetheless, as the measurement strategy for this variable will not be applied differentially among the groups, in the event of a bias, the association measure would tend to be null.

Additionally, the applicability of the study results may be altered by the scarcely predictable epidemiological behavior of dengue (whether endemic or epidemic), depending on the moment sampling kicks off. Accordingly, data collection will commence during the second quarter of the year - a period considered endemic based on the observations of previous years (Figure 2)--such that the findings of the project may prove applicable both in endemic-transmission periods and at the beginning of outbreaks.

Lastly, it has been established that dengue incidence is directly proportional to the emergence of acute cases and related deaths. Hence, knowing where the transmission chain commences would make it possible to focus the most successful control actions in such a manner that they exert an impact on halting transmission, thereby effectively quelling the dengue attack and mortality rates. In this regard, the present study will contribute valuable information towards clarifying whether the peridomestic areas are in fact the starting point of transmission. Should the contrary be revealed, research should be redirected to studies which explore sites other than the subjects' dwellings for the occurrence of infection.

## Abbreviations

DALYs: Disability-adjusted life years; DENV: Dengue virus; HI: House index; SSM: Servicios de Salud de Morelos; IC: Index case; RR: Relative risk; NS1: Nonstructural protein 1; RT-PCR: Reverse transcription polymerase chain reaction; ELISA: Enzyme linked immunosorbent assay; IgM: Immunoglobin M; IgG: Immunoglobin G; GPS: Global positioning system.

## Competing interests

The authors declare that they have no conflicts of interest with sponsorship in the elaboration of this contribution.

## Authors' contributions

All the authors participated not only in the elaboration of the present research protocol, but also in the revision and approval of the final version of the manuscript.

## Pre-publication history

The pre-publication history for this paper can be accessed here:

http://www.biomedcentral.com/1471-2458/12/262/prepub
